# Interrelationship Between Intelligence Quotient and Space Maintainers Among Children: A Cross-Sectional Comparative Study

**DOI:** 10.7759/cureus.50752

**Published:** 2023-12-18

**Authors:** Ayesha Fathima, Ganesh Jeevanandan

**Affiliations:** 1 Pediatric and Preventive Dentistry, Saveetha Dental College And Hospitals, Saveetha Institute of Medical and Technical Sciences, Saveetha University, Chennai, IND; 2 Pediatric and Preventive Dentistry, Saveetha Dental College and Hospitals, Saveetha Institute of Medical and Technical Sciences, Saveetha University, Chennai, IND

**Keywords:** cognitive effects, pediatric oral health, pediatric dentistry, intelligent quotient, space maintainers

## Abstract

Introduction

Intelligence quotient (IQ) is an indicator to measure a child's cognitive ability to learn or understand and to deal with new situations with their logical and analytical skills. Children with better IQ exhibit increased cooperation when undergoing dental treatments, leading to a positive attitude toward dental care. The primary aim of the study was to assess the interrelationship between the IQ of children, space maintainer therapy, and the behavior of children aged 6-10 years.

Materials and methods

A total of 104 children were divided into two groups: group 1 included children undergoing space maintainer therapy and group 2 included children who did not undergo space maintainer therapy. Their IQ scores were assessed using Raven’s Coloured Progressive Matrices and behavior and the Frankl behavior rating scale. The data were analyzed by SPSS Version 23 (IBM Corp., Armonk, NY). Independent t-tests were used to evaluate the differences between IQ and children with space maintainers, and Mann-Whitney U tests were used to assess the differences between behavior and space maintainers.

Results

The mean age of the participants was approximately 8.28 years. The mean IQ score of the group of children undergoing the space maintainer therapy was 90.69 ± 7.65 and that of the control group was 105.59±10.71. Based on the Frankl behavior rating scale, the mean score in the space maintainer group was 35.44 and that of the control group was 69.56. There was a significant association between IQ, behavior, and the presence of space maintainers.

Conclusion

The group of children undergoing space maintainer therapy demonstrated comparatively lesser IQ, and the majority of children exhibited negative behavior. Also, children wearing space maintainers had undergone one or multiple extractions, which is traumatic for children and may lead to them likely exhibiting a negative behavior than children in the control group. Hence, it may be concluded that intelligence, behavior, and space maintainers are all significantly associated with each other.

## Introduction

One of the greatest challenges in pediatric dentistry is the management of space loss due to untimely loss or early exfoliation of primary teeth. However, we often see cases in which extraction of primary teeth becomes a definitive treatment due to grossly carious tooth, irreversible pulpitis with poor prognosis, trauma, or iatrogenic causes [1]. In such cases, discrepancies in the arch length can undergo drastic changes, leading to malocclusion, impaction, and supra eruption of the opposing tooth during the mixed or permanent dentition phase [2]. The easiest management to prevent this frequently encountered issue is to retain the primary teeth in the oral cavity until they exfoliate, as the primary dentition plays a vital role in the eruption guidance of its successor teeth. In case of discrepancy in the arch length, a space maintainer has to be provided to maintain the arch length [3]. Studies have proven that oral health diseases and the intelligence quotient (IQ) of children have a significant association, as it is seen that IQ is a strong predictor of a child’s cognitive ability [4,5].

IQ is the comparative measure of a person’s intelligence, represented by a score obtained from a standardized intelligence test [6]. It may also influence their ability to communicate feelings of distress and to behave adequately in the dental situation [7]. The Wechsler Intelligence Scale for Children (WISC) [8,9] and Raven's Coloured Progressive Matrices (RCPM) are two commonly used tools that measure the IQ of children and adults [10,11]. Various factors such as development and personal experiences play a role in shaping an individual's intelligence over time [12]. In the current study, fluid intelligence and reasoning ability are widely applied interchangeably. The level of intelligence determines the outcome of dental treatment since dentistry is an interactive field [13]. Henceforth, RCPM is used to evaluate the IQ of children in this study; it is designed to measure the level of both intellectual development and logical thinking due to its simplicity in questioning. Children's perception of causes and effects, information, and instructions, as well as their attitude toward dentistry, can all be expected to be significantly altered by their level of intelligence. Also, it affects how they can express their emotions and behave appropriately in dental situations [14]. Due to their perceptive levels, children with lower IQs sometimes express negative behavior. On the other hand, during dental operations, children with above-average IQs and children with outstanding IQs both show favorable degrees of collaboration and positive cooperation [15]. Therefore, a child's oral health, intelligence, and behavior are all interrelated. However, the scarcity of literature support on this topic mandates the need for the present study.

The primary aim of the study was to determine the association between IQ, children’s behavior, and the presence of space maintainers in children aged 6-10 years in a private dental setting.

## Materials and methods

This cross-sectional comparative study was carried out in the outpatient department of pediatric and preventive dentistry at a private dental institution in Chennai, Tamil Nadu, India, which included patients in the age group of 6-10 years. A total of 104 children were divided equally into two groups: group 1 (test group) included children undergoing band and loop space maintainer therapy in the mandibular region and group 2 (control group) included children who did not undergo any space maintainer therapy based on a convenient sampling method.

Inclusion criteria

Participants who were medically fit, free of any chronic systemic illness, and living with their respective family members were included in the study. Participants selected were free of any developmental disorders associated with psychiatric illness. All the participants in the study were from the same socioeconomic background and geographical region.

Exclusion criteria

Participants or parents who did not give consent to participate in the study, those who required special health care needs, and those with a history of any oral habits such as thumb sucking or bruxism, which may have psychological etiology associated with it, were excluded.

Before beginning the study, ethical clearance from the Scientific Review Board was obtained from the university where the study was conducted (ethical clearance number: SRB-2104/22/036). Written informed consent was acquired from the parent/guardian of the participants. To protect the participants’ privacy, their identities were kept anonymous throughout the study. Data collection was scheduled for the months of February to April 2023. The purpose of the research was explained to the parents. Data were collected using two questionnaire forms. One of them is a logical test questionnaire, in which the child can identify answers and the answers were filled by the pre-trained dentists in the department. Another questionnaire was also filled out by the same pre-trained dentist after observing the child. The World Medical Association Declaration of Helsinki's guiding principles were strictly followed throughout the study's execution. According to the study [5], with a p-value of 0.05% and 95 power with an effect size of 0.636, the sample size was calculated using G power software. The estimated sample size was 104 participants.

Survey instrument

**Figure 1:**
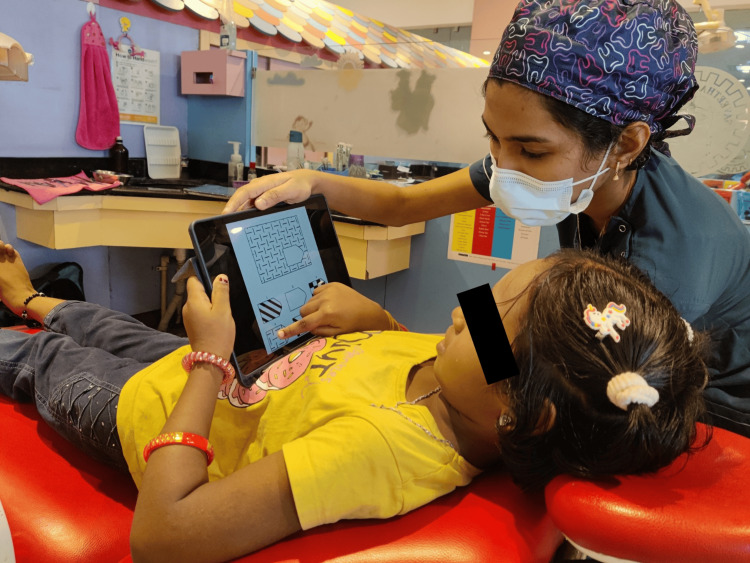
A participant attending the logical test questionnaire using the protocols outlined in Raven's Coloured Progressive Matrices under the supervision of a trained pediatric dentist.

RCPM was used to determine each child's intellectual capacity. Matrix representations of the models were shown. The child was asked to choose the component that was lacking from each test item in order to complete the model. The test was conducted using the protocols outlined in RCPM within the department under the supervision of a trained pediatric dentist (Figure 1).

The questionnaire was finished on average in 30 minutes. The level of difficulty on the test ranged from simple to challenging. Each issue had a geometric matrix with six choices for each deleted cell, but only one of the alternatives really fit. According to the current Wechsler categorization, children's IQ scores were split into six groups: superior (120-129), high average (110-119), average (90-109), low average (80-89), borderline (70-79), and severely low (70) [8,9]. The second questionnaire was the Frankl Behavior Rating Scale (FBRS) [16,17], which divides children's behavior in dental chairs into four categories: definitely negative, negative, positive, and definitely positive [18].

Statistical analysis

Cumulative data were collected and analyzed using the SPSS software Version 23 (IBM Corp., Armonk, NY). The analysis used descriptive statistics with a 95% confidence interval including frequency and percentage for gender, as well as mean and standard deviation for age. The chi-square test was used to assess the association between FBRS and the presence of space maintainers and between IQ score groups and the behavioral scale, and the independent t-test was used to assess differences in means of IQ score and the presence of space maintainers across groups. Additionally, at a significance level of p<0.05, the Mann-Whitney U test was used to evaluate differences between the presence of space maintainers and behavior rating scale.

## Results

**Figure 2:**
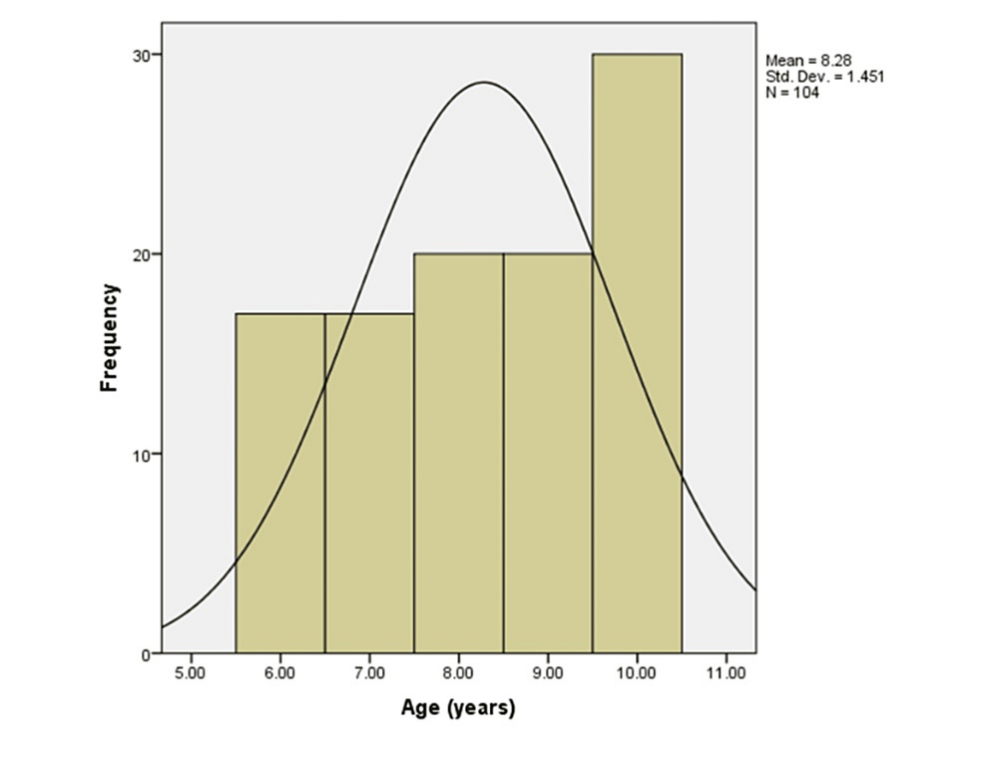
Age distribution of the study participants

The study consisted of 104 participants divided into two groups, with 52 participants in each group. Of these, 57 were females and 47 were males. Figure 2 shows that the average age of the participants was 8.28 years, with a standard deviation of 1.45 years.

The mean IQ score of the total population was 98.14 ± 11.9. The IQ scores in each group were distributed as follows. In group 1 (participants with space maintainer), two participants had “high average,” 23 participants had “average,” 25 participants had “low average,” and two participants had “borderline” IQ scores. In group 2 (control group), 10 participants had “superior,” 10 participants had “high average,” 29 participants had “average,” and three participants had “low average” IQ scores (Table 1).

**Table 1:** Distribution of IQ scores of the space maintainer group and the control group

Group	Score groups	Number of participants
Space maintainer group	High average	2
	Average	23
	Low average	25
	Borderline	2
	Total	52
Control group	Superior	10
	High average	10
	Average	29
	Low average	3
	Total	52

The behavior of children was measured according to FBRS, ranging from definitely negative to definitely positive. In group 1, two participants had “definitely negative,”, 24 participants had “negative,” 13 participants had “positive,” and 13 participants had “definitely positive” behavior toward the treatment. In group 2, three participants had “negative,” 27 participants had “positive,” and 22 participants had “definitely positive” behavior toward their dental treatment (Table 2).

**Table 2:** Distribution of behavior rating scale scores of the space maintainer group and the control group

Group	Score groups	Number of participants
Space maintainer group	Definitely negative	2
	Negative	24
	Positive	13
	Definitely positive	13
	Total	52
Control group	Negative	3
	Positive	27
	Definitely positive	22
	Total	52

The data were analyzed for normality with Kolmogorov-Smirnov test and proved to be normally distributed. Pearson’s chi-square tests revealed that there is a significant association between the presence of space maintainers, children’s behavior, and IQ scores (Tables 3-5).

**Table 3:** Chi-square test showing a significant association between IQ score groups and the presence of space maintainers among the study participants *Significant (p < 0.05)

Presence of space maintainers	IQ score groups						
	Superior	High average	Average	Low average	Borderline	Total	p-value
Space maintainer group	0	2	23	25	2	52	0.0002*
Control group	10	10	29	3	0	52	
Total	10	12	52	28	2	104	

**Table 4:** Chi-square test a showing significant association between the Frankl behavior scale and the presence of space maintainers among the study participants *Significant (p < 0.05)

Presence of space maintainers	Frankl’s behavior scale					
	Definitely negative	Negative	Positive	Definitely positive	Total	p-value
Space maintainer group	2	24	13	13	52	0.0006*
Control group	0	3	27	22	52	
Total	Total	27	53	22	104	

**Table 5:** Differences in the distribution of IQ scores in the space maintainer group and the control group *Significant (p < 0.05)

IQ scores and the presence of space maintainers	Mean ± SD	p-value
Space maintainer group	90.69 ± 7.65	0.0004*
Control group	105.59±10.71	

The independent t-test was used to compare IQ scores between the groups and yielded statistically significant differences between each other, with the control group having higher IQ scores (Table 6).

**Table 6:** Chi-square test showing significant association between frankl’s behaviour scale and IQ score groups among the study participants *Significant (p < 0.05)

Frankl’s behaviour scale	IQ score groups						
	Superior	High average	Average	Low average	Borderline	Total	p-value
Definitely negative	0	0	0	0	2	2	0.0006*
Negative	0	0	7	20	0	27	
Positive	0	4	41	8	0	53	
Definitely positive	10	8	4	0	0	22	
Total	10	12	52	28	2	104	

Also, the Mann-Whitney U test was used to compare the behavior between the groups and showed that the control group had significantly higher “positive” and “definitely positive behavior” than the space maintainer group (Table 7).

**Table 7:** Differences in the distribution of behavior in the space maintainer group and the control group *Significant (p < 0.05)

Presence of space maintainers and Frankl’s behavior scale	Mean rank	Mann-Whitney U value	p-value
Space maintainer group	35.44	465.000	0.0006*
Control group	69.56		

## Discussion

IQ is defined as the relative intelligence of an individual expressed as a score on a standardized test of intelligence. There are various IQ testing scales, such as the Stanford-Binet IQ test, Kaufman Tests, Cognitive Assessment System, Differential Ability Scales, Sternberg Triarchic Abilities Test, Turing test, Wechsler Intelligence Scale, and RCPM. RCPM is a commonly implemented mode of IQ testing in educational contexts, in contrast to other IQ testing scales [11]. Moreover, individuals with higher intelligence tend to understand the causes and effects of dental issues better, comprehend information and instructions effectively, and, as a result, are expected to behave appropriately and communicate more comfortably in a dental clinic setting [19]. The present study used RCPM, which is designed for normal, intellectually and physically impaired children aged 5-11 years [5,20]. The IQ scores in the control group were found to be significantly higher compared to the space maintainer group. This can be explained as children with more dental caries or more DMFT (sum of the number of Decayed, Missing due to caries, and Filled Teeth in the permanent teeth) scores had significantly lesser IQ scores than those who had less dental caries in the teeth that were not indicated for extraction [21].

Similar results were obtained in the study conducted in India, where researchers examined the connection between IQ scores and dental caries and discovered a statistically significant correlation between the two groups [22]. A substantial correlation between dental caries, fluorosis, and IQ scores has been shown in similar studies [23]. Similar results were obtained in the studies conducted in Thailand and Istanbul, which found associations of sociodemographic and behavioral factors with DMFT and condition-specific impacts attributed to dental caries, respectively [24,25].

According to the literature, individuals with higher levels of intelligence exhibit lower occurrences of dental caries and a reduced amount of dental plaque compared to those with lower intelligence levels [5]. This suggests that higher intelligence correlates with increased awareness of dental care, including both preventive measures and dental problem treatment [26].

Limitations

The study included a limited number of participants, and the study design was cross-sectional. The etiology in the development of a child's IQ and behavior is multifactorial. However, this study included a very narrow spectrum of participants who had undergone space maintenance therapy. The study did not exclude children who had a previous negative dental experience, and parental attitude was not considered during study. There is no follow-up for the study to analyze if there is any behavioral change toward the dentist. Accessibility to dental care is not included and analyzed. Contributing factors for changes in behavior or IQ, such as personal or emotional development of the child, nutrition, or type of diet, were not considered in the present study.

## Conclusions

The present study within its limitations concludes that children who do not require a space maintainer have low DMFT scores and comparatively higher IQ than children undergoing space maintainer therapy. Also, children wearing space maintainers had undergone one or multiple extractions, which may have led them to likely exhibit negative behavior than children in the control group. Hence, it may be concluded that intelligence, behavior, and space maintainers are all significantly associated with each other.
